# Sodium channel blockers as add-on treatment for unexplained refractory chronic cough: a case report and review

**DOI:** 10.3389/fmed.2025.1742373

**Published:** 2026-01-12

**Authors:** I. Butnariu, M.-F. Balan, D. C. Zaharia, R. Bobocea, Iulia Ana-Maria Mitrică, A. Moraru, V. Dionisie, Vlad-Iulian Lăptoiu, D. Antonescu-Ghelmez, F. Antonescu

**Affiliations:** 1 Department of Clinical Neurosciences, Carol Davila University of Medicine and Pharmacy, Bucharest, Romania; 2 Neurology Department, National Institute of Neurology and Neurovascular Diseases, Bucharest, Romania; 3 Department of Pneumology I, Carol Davila University of Medicine and Pharmacy, Bucharest, Romania; 4 Pneumology Department, “Marius Nasta” Institute of Pneumology, Bucharest, Romania; 5 Department of Psychiatry and Psychology, Carol Davila University of Medicine and Pharmacy, Bucharest, Romania; 6 Department of Adult Psychiatry, “Prof. Dr. Alexandru Obregia” Clinical Hospital of Psychiatry, Bucharest, Romania

**Keywords:** carbamazepine, chronic cough, cough hypersensitivity syndrome, internal jugular vein aneurysm, internal jugular vein ectasia, primary chronic cough, sodium channel blockers, unexplained chronic cough

## Abstract

**Introduction:**

Chronic cough is a common symptom with multiple causes, predominantly respiratory, gastroesophageal, or neurological. However, in rare cases, a vascular anomaly, such as internal jugular vein ectasia (IJVE), may function as a trigger. We report the case of a patient with paroxysmal chronic cough associated with right internal jugular vein ectasia, which responded favorably to anticonvulsant therapy.

**Case report:**

We present the case of a 62-year-old female patient with pulmonary, cardiovascular, and neurological comorbidities, with a two-year history of daily episodes of severe, non-productive cough, refractory to standard symptomatic treatments. ENT, pulmonary, and gastroenterological evaluations did not identify a specific organic cause. The neurological examination was unremarkable, with no cranial nerve deficits. Cervical CT with contrast revealed a fusiform right internal jugular vein ectasia (IJVE) (area of 2.11 cm^2^ compared to the left side, 0.50 cm^2^). A chronic neurogenic cough possibly linked to the IJVE was suspected, and treatment with carbamazepine (300 mg three times daily) and codeine (15 mg four times daily) was successful in near-complete cough suppression.

**Conclusion:**

This case adds to the emerging evidence regarding a possible link between IJVE and chronic neuropathic cough. Although rare, this etiology should be considered in chronic cough syndromes without an apparent cause. Neuropathic cough is underdiagnosed and may benefit from anticonvulsant treatment, while the identification of atypical mechanisms requires a multidisciplinary approach.

## Introduction

Chronic cough is a prevalent and challenging clinical symptom, defined as a cough persisting for more than 8 weeks (1). While it is most commonly associated with conditions such as gastroesophageal reflux disease (GERD), upper airway cough syndrome, asthma, and the use of ACE inhibitors, up to 40% of patients referred to specialty clinics present with unexplained or refractory chronic cough (2).

Recent research increasingly indicates that neurogenic factors contribute to at least some cases. Several case reports have proposed an association between internal jugular vein ectasia (IJVE), a rare and usually asymptomatic vascular anomaly, and episodes of intractable or paroxysmal cough. It is hypothesized that mechanical compression or irritation of adjacent neural structures, particularly the vagus nerve or its branches, may play a role in eliciting the cough reflex (3–5).

This article examines the clinically significant association between refractory chronic cough (RCC) and IJVE, synthesizing evidence from current literature regarding this rare condition, as well as the potential therapeutic efficacy of the sodium channel blocker carbamazepine.

## Case report

A 62-year-old woman was referred to our clinic for neurological evaluation due to a two-year history of isolated, severe, spastic, nonproductive cough of unknown etiology. The episodes occurred almost daily, with individual events sometimes lasting up to 30 min, significantly impairing respiratory function and frequently resulting in vomiting. Occasional episodes woke the patient from her sleep. These episodes arose spontaneously, without identifiable triggers, without hoarseness or dysphonia. Only occasionally, the cough was preceded by a “tickling” sensation in the throat. There was no preceding history of trauma or infection, except for a mild case of COVID-19 3 months prior to symptom onset. She scored 7.1 points on the Weekly Cough Severity Diary (WCSD).

*The patient’s medical history* was notable for well-controlled arterial hypertension (treated with indapamide, candesartan, and nebivolol), dyslipidemia (managed with rosuvastatin and ezetimibe) and Hashimoto’s thyroiditis with hypothyroidism (for which she received levothyroxine). Additional comorbidities included previous surgeries for C4-C5 and L5-S1 disc herniation, as well as a total hysterectomy. Notably, she experienced cardiorespiratory arrest during two separate surgical procedures—one for hysterectomy in 1998 and one for cervical disc herniation in 2011.

The search for the etiology of the patient’s cough started in the cardiology department. She had initially been treated for systemic hypertension with a combination of perindopril/indapamide and bisoprolol. Given the well-documented association between ACE inhibitors and cough and considering that beta-blockers can also exacerbate cough in susceptible individuals, the cardiology team had replaced perindopril and bisoprolol with candesartan, indapamide, and nebivolol (6, 7). However, this change did not result in any improvement in her symptoms.

Over the course of 2 years, the patient had undergone an extensive multidisciplinary evaluation, including repeated consultations in cardiology, ENT, pneumology, gastroenterology, allergology, endocrinology, surgery, and psychiatry. For an exhaustive list see Supplementary Table 1.

Pneumological evaluations of possible pulmonary/respiratory causes of chronic cough (lung tumors, bronchiectasis, asthma, allergic alveolitis, etc.). Multiple CT scans, with and without contrast enhancement, were unremarkable. Bronchoscopy showed only mild, non-specific inflammatory changes of the mucosa at the level of the hypopharynx and larynx, possibly secondary to severe coughing but also possibly related to GERD. Spirometry showed a normal Tiffeneau index (84%), normal forced expiratory volume in the 1st second (FEV1 2,61 L – 117% vs. predicted value) and normal forced vital capacity (FVC 3.1 L – 110% vs. predicted value). Microbiological testing from bronchial aspirate, including GeneXpert for tuberculosis, fungal, and bacterial cultures, was negative.

Gastroenterological assessment (mostly for GERD suspicion), including upper gastrointestinal endoscopy and barium swallow, identified a small hiatal hernia, minor gastroesophageal reflux, and erythematous antral gastritis, none considered of significance in the context. Esophageal manometry confirmed the presence of a hypersensitive esophagus.

A psychiatric evaluation ruled out any specific disorders and considered a psychogenic etiology highly unlikely.

Treatment with proton pump inhibitors, baclofen, codeine and other symptomatic agents failed to alleviate the cough. Additional therapies, including antihistamines, macrolide antibiotics, and both oral and inhaled corticosteroids, were also ineffective (Supplementary Table 1) raising the suspicion that the cough had not a common cause and the patient was referred to neurology.

At presentation in the neurology department the general clinical and neurological examination, including cranial nerve assessment, revealed no abnormalities.

**Figure 1:**
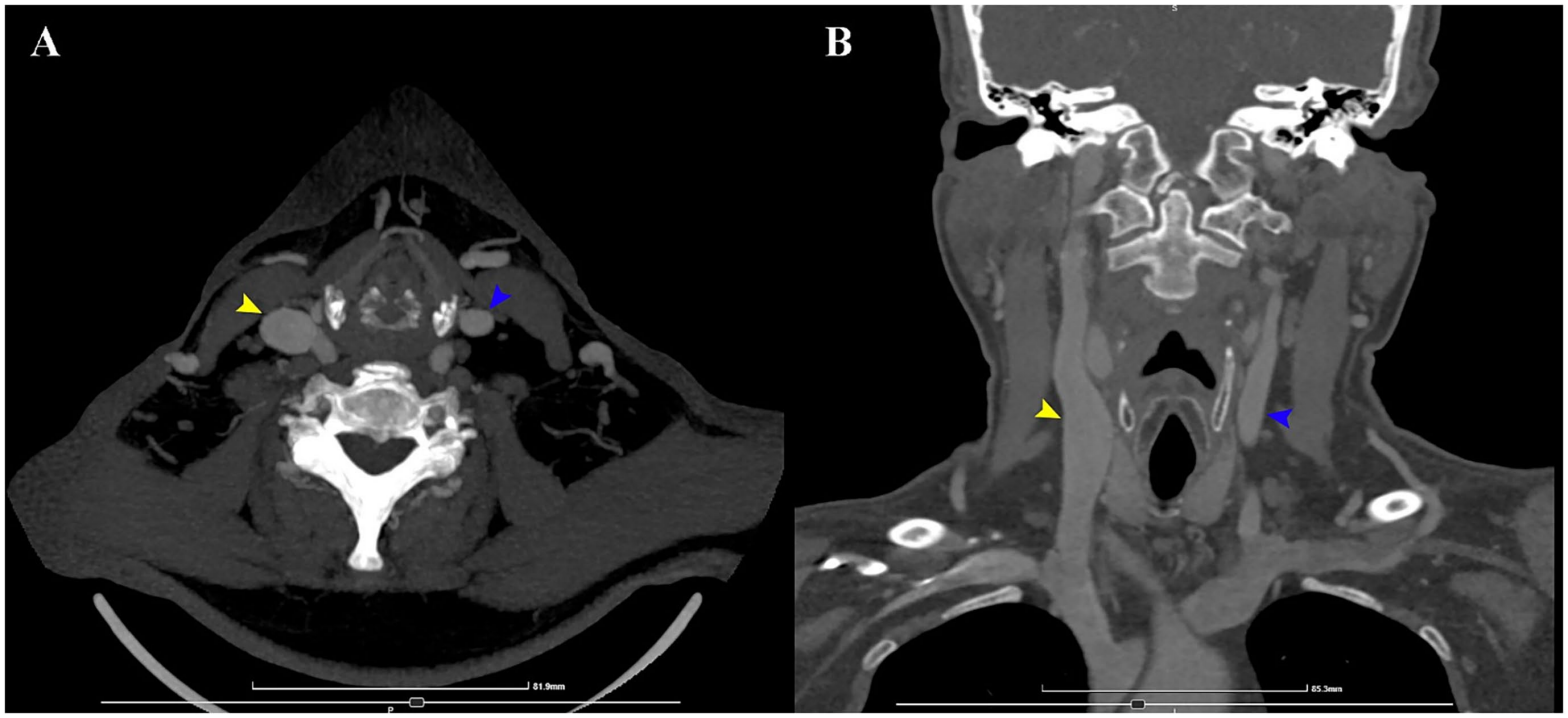
CT angiography of the neck axial **(A)** and coronal **(B)** sections. The internal jugular veins are visible (yellow, respectively blue arrows). The maximum transverse and anteroposterior diameters of the internal jugular veins were 1.53 cm and 1.75 cm on the right, and 0.87 cm and 0.72 cm on the left, respectively. The corresponding cross-sectional areas were approximately 2.11 cm^2^ on the right and 0.50 cm^2^ on the left.

Routine laboratory testing was within normal limits, apart from an elevated anti-thyroid peroxidase antibody level. Vasculitis and infectious disease panels were negative. Brain MRI revealed only mild cerebral small vessel disease. Cervical spine MRI demonstrated significant degenerative changes, without signal abnormalities in the cervical or upper thoracic spinal cord. Further evaluation with contrast-enhanced cervical CT revealed fusiform ectasia of the right internal jugular vein, with a cross-sectional area of approximately 2.11 cm^2^ compared to 0.50 cm^2^ on the contralateral side (Figure 1). This anomaly prompted the medical team to search the literature for similar case reports.

Given the suspected neurogenic chronic cough and possible vascular irritation of the vagus nerve (as the literature suggests in similar cases), treatment with carbamazepine was initiated at 400 mg/day (200 mg bid) for 14 days, subsequently increased to 600 mg daily (300 mg bid). At the three-month follow-up, the patient reported a subjective reduction by one third in both the frequency and intensity of the coughing episodes, although she continued to experience occasional severe attacks. WCSD improved to 5.2 points. Following the addition of codeine at a dose of 60 mg per day (15 mg qid) and an increase in carbamazepine to 900 mg daily (450 mg bid), the patient experienced prompt improvement with at least a 50% reduction in both frequency and severity of symptoms. At the one-month follow-up, she reported complete resolution of the cough, having remained symptom-free throughout the previous month. At the 6 months follow-up, she reported only slight coughing in some of the days, with many days free of coughing, with only 2 severe attacks in the last 3 months, spaced about a month apart. She reported no side effects from carbamazepine, and some sedation and dizziness from codeine. Attempts to gradually taper codeine were unsuccessful, as symptoms reappeared whenever the dose dropped below three tablets per day; these symptoms subsided once the previous dosage was reinstated. Carbamazepine has not been tapered yet, as the patient is reluctant to try, due to concerns about cough relapse and the perceived benefit it provides compared to codeine alone. Longer follow-up is needed.

**Figure 2:**
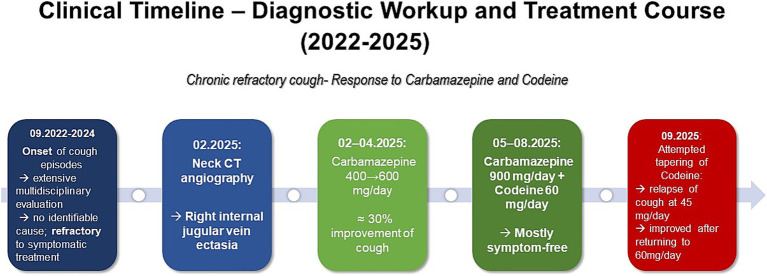
Clinical timeline summarizing diagnostic evaluation and treatment course of a patient with chronic refractory cough, showing progressive improvement under carbamazepine and codeine therapy.

A visual summary of the patient’s diagnostic workup and treatment course is shown in Figure 2.

## Discussion

### Difficulties in framing the diagnosis

The current expert consensus classifies persistent cough without a known cause, after completion of investigations according to published guidelines, as unexplained chronic cough (UCC) or unexplained refractory chronic cough (URCC). If a cause is subsequently identified, cases may be reclassified as explained refractory chronic cough (ERCC) (8, 9). This classification has limitations in both clinical and research contexts, as it obviously says not much besides that we are facing the unknown, and patients are understandably dissatisfied with such labels.

To address this gap, Cough Hypersensitivity Syndrome (CHS) has recently been considered as a possible underlying factor in many cases (8). CHS aims to be defined as a hyperactive cough reflex, which in itself is no simple thing to describe, characterized by exaggerated responses to cough-provoking stimuli (hypertussia), as well as cough triggered by ordinarily innocuous stimuli, such as talking, laughing, eating (allotussia) (10). As currently framed, CHS extends to include patients with recognized etiologies who nonetheless exhibit heightened reflex sensitivity, an aspect which can dilute its practical utility.

Most recently (2025), Song et al. proposed revising the current paradigm, which considers cough primarily as a symptom, by suggesting primary chronic cough (PCC) as a distinct entity featuring cough reflex hypersensitivity as a treatable trait, analogous to the “primary chronic pain” concept (11). This model implies a neurogenic mechanism, sometimes associated with chronic mechanical vagal irritation or other causes of vagal neuropathy (e.g., viral, autoimmune origins), while in other cases, a transient ENT or pulmonary event triggers sustained upregulation of the reflex (10, 12). Evidence from cerebral functional MRI studies appears to support this hypothesis (13).

Our patient fits both URCC and PCC but lacks the typical symptoms of CHS.

### Current evidence for neuromodulator therapies

A 2018 review of neuromodulatory treatments for “chronic cough”—broadly aligning with what is now termed URCC—framed cough-reflex hypersensitivity as a plausible mechanism and found evidence for positive effects of gabapentin, pregabalin, amitriptyline, and the P2X3 antagonist gefapixant (14).

A subsequent 2023 systematic review and meta-analysis targeting “chronic airway hypersensitivity” (CAH)—an umbrella construct encompassing CHS, sensory neuropathic cough, post-viral vagal neuropathy, irritable larynx syndrome, neurogenic cough, laryngopharyngeal neuropathy, and laryngeal sensory neuropathy, and broadly aligning with UCC—was able to include only three studies (15). These evaluated gabapentin, eliapixant, and gefapixant and demonstrated improvements in patient-reported cough severity, with little or no reduction in objective cough frequency—a pattern consistent with the 2018 review (14, 15). Similarly, in our case, it was the severity of the attacks that primarily contributed to discomfort, rather than their frequency.

There are two current guidelines available for the management of chronic cough, the CHEST Guideline and Expert Panel Report from 2016, respectively the European Respiratory Society (ERS) Chronic Cough Guidelines, published in 2020. According to the mentioned available evidence, pregabalin and, particularly, gabapentin are recommended in both publications for the management of RCC (16, 17).

Carbamazepine appears in the chronic cough literature in only isolated and generally old reports (18, 19).

### The internal jugular vein ectasia and possible conflict

IJVE is an uncommon vascular condition that is frequently identified incidentally during imaging or as a neck swelling that becomes more prominent with actions such as talking, coughing, or swallowing. The definition of ectasia involves a venous diameter that is 2 to 3 times larger than the contralateral normal vein, or an absolute vessel diameter greater than 15 mm (20, 21). While it is most often recognized in children, cases of IJVE appearing in adults—predominantly in women—have also been documented and are typically unilateral, with a right side preference (4).

The prevalence of asymptomatic IJVE in the general adult population remains unknown, as the literature is sparse. In a systematic review from 2019 only 41 adult cases were identified worldwide, emphasizing the rarity of this vascular anomaly in adults (4). These findings are consistent with a more recent pooled analysis from 2022, which similarly found that adult cases are markedly underrepresented when compared with pediatric cohorts (22).

In the reviewed literature, most patients were asymptomatic, typically presenting with a painless, Valsalva-dependent cervical swelling—a feature not observed in our patient. Symptomatic presentations were reported only rarely and included voice changes, neck pain, chest discomfort, dysphagia, Horner syndrome, or chronic cough. Among these, cough accounted for less than 2% of all cases, while pain was the most frequently described symptom, at approximately 12% (4, 22, 23).

The underlying causes are not fully established; however, both congenital factors, such as decreased venous wall elasticity, and acquired factors, including neck trauma, increased intrathoracic pressure, or prior catheterization, have been suggested (4, 24). Right-sided predominance may be related to structural characteristics: the right internal jugular vein has a higher valve position, larger diameter, and more direct connection to the superior vena cava compared to the left. These anatomical differences can facilitate the transfer of intrathoracic pressure to the jugular bulb, contributing to localized dilation (4).

The concept of a neurovascular conflict, in which a blood vessel compresses or irritates a cranial nerve, leading to hyperexcitability or dysfunction has occasionally been described in conjunction with the vagus nerve (25). A particularly relevant example is VANCOUVER syndrome, which describes a chronic neurogenic cough resulting from vascular compression of the vagus nerve at its root entry zone (26). Another vascular-nerve interaction involving the internal jugular vein is Vernet syndrome, classically characterized by simultaneous involvement of cranial nerves IX, X, and XI at the jugular foramen. Interestingly, the persistent cough itself was proposed as a possible contributor to the onset of Vernet syndrome due to mechanical stretching or irritation in the region of the jugular foramen (5).

The mechanisms are not yet fully understood, but possible ways in which IVJE could impact the vagus nerve and sustain cough include stretching of the nerve during neck movements or Valsalva maneuvers, turbulent venous flow within the carotid sheath, and prolonged pressure from the ectatic vein (3, 4).

### Making our case

The patient underwent comprehensive evaluations across multiple specialties, which failed to identify a cause. The lack of response to conventional therapies led to consideration of a neurogenic origin. Neurological examination was normal, and brain and spine imaging was unremarkable. Fusiform ectasia of the right internal jugular vein was identified as a possible anatomical factor capable of inducing a peripheral vagal conflict. An additional aggravating factor to consider is that coughing elevates pressure within the jugular system, which may perpetuate, in a positive feedback fashion, nerve irritation and subsequently maintain the cough reflex, potentially accounting for the prolonged duration of these episodes.

Based on the hypothesis of the sensory neuropathic cough and lack of response to all previous treatments, the patient was tentatively started on carbamazepine, which has been reported previously to reduce cough by potentially modulating afferent vagal nerve activity (26–28). The clinical response was good, and significant improvement followed further after adding codeine, which had had had no effect in monotherapy previously, even at larger doses that the ones used currently. Longer follow-up is needed to monitor for adverse reactions and the possibility of relapse.

Carbamazepine is a sodium-channel blocker that stabilizes inactivated channels and preferentially inhibits high-frequency neuronal firing, with apparent effectiveness in cases of focal peripheral nerve hyperexcitability (29–32). Gabapentinoids bind to the α2δ-1 subunit of voltage-gated calcium channels, leading to reduced presynaptic glutamate release, which is more relevant for central sensitization and persistent neuropathic pain (33, 34).

## Conclusion

To the best of our knowledge, this is the second case of chronic cough in adults possibly caused by internal jugular vein phlebectasia and the third if the pediatric population is included (3, 5).

We hope our report contributes to expanding the limited body of literature that suggests a potential association between vascular anomalies and chronic cough. While the causal relationship between IJVE and chronic cough syndromes has not been definitively established, it is noteworthy that sodium channel blocker treatment yielded a positive therapeutic response. This observation, comparable to outcomes seen in trigeminal neuralgia and hemifacial spasm, may suggest an analogous vagus nerve involvement. Ongoing follow-up remains necessary for monitoring the risk of potential relapse.

Vascular anomalies in the cervical region are not emphasized in protocols for investigating UCC, such as those issued by the ERS (17). We believe that screening the cervical course of the vagus nerve for vascular or other structural conflicts could be beneficial in such patients and may provide useful guidance for treatment.

Neuropathic pain is recognized for its intricate and multifactorial mechanisms, each providing distinct avenues for therapeutic intervention (31). If we are to extend this comparison to neuropathic cough we can assume the same level of complexity. Gabapentinoids are already present in the current treatment guidelines for URCC. We propose there is a rationale for considering sodium channel blockers, such as carbamazepine or oxcarbazepine, as viable treatment options, particularly in situations involving suspected vasculo-nervous conflict or where gabapentinoids have proven ineffective.

### Patient’s perspective

For the last 2 years, I suffered from a persistent and severe cough that resisted treatment despite multiple specialist consultations and many investigations. After other options failed, I was referred to the Institute of Neurology, where a dedicated medical team managed my therapy and has succeeded in significantly improving my condition. By August, my symptoms had greatly diminished, with only one notable episode in 2 months. I am grateful for their care, which has allowed me to return to a normal life.

## Data Availability

The original contributions presented in the study are included in the article/Supplementary material, further inquiries can be directed to the corresponding author.
