# Supplementary material for: Sodium channel blockers as add-on treatment for unexplained refractory chronic cough: a case report and review
Source: Front Med (Lausanne). 2026 Jan 12;12:1742373. doi: 10.3389/fmed.2025.1742373 (PMC12833317; doi:10.3389/fmed.2025.1742373)
Supplement: Supplementary file 1 [file Data_Sheet_1.pdf]

Supplementary Table 1. Summary of multidisciplinary evaluation, treatments, and outcomes in chronological order.

Date and evaluation	Investigation	Findings	Treatment	Outcome
**09.2022**			Dietary supplement (Astha-15) 3 weeks	Ineffective
**10.2022 Pneumology**	Chest X-ray<br>Spirometry	No Rx lesions<br>Normal spirometry	Amoxicillin / Clavulanic acid 1g twice/daily- 10 days<br>Herbal medicinal product<br>Followed by Pantoprazole 40mg/daily<br>Intranasal corticosteroid (Beclometasone dipropionate 1 spray twice daily)	No clear improvement
**10.2022 ENT**	ENT fibroscopic examination	Postnasal drip syndrome		
**11.2022 Pneumology**	1.Chest CT<br>2.DLCO<br>3.Comprehensive pulmonary function tests (PFTs)	1. Mild cylindrical bronchiectasis in the bilateral lower lobes<br>2,3. Normal values	Inhaled corticosteroid (Fluticasone propionate, 1 spray bid for 7 days)<br>Mucolytic therapy (Erdosteine 300mg, 2 tables once daily for 7 days)<br>Antihistamine therapy (Bilastine20 mg, 1 tablet once daily for 7 days)<br>Codeine 15mg, 1 tablet four times daily for 3 weeks	No improvement<br>No improvement
**12.2022 Gastroenterology**	Upper GI endoscopy	Hiatal gastric hernia approx. 5 cm, antral erythematous gastritis, possible GERD	Pantoprazole switched to Rabeprazole 20 mg, 1 tablet twice daily for 2 months	No improvement
**12.2022 ENT**	Throat swab	Negative for streptococcus, staphylococcus, fungi, candida and Bordetella pertussis.		
**01.2023 Gastroenterology**	High-resolution esophageal manometry	Hypotonic lower esophageal sphincter (LES) with normal	Continue Rabeprazole 20mg, 1 tablet bid<br>Added Baclofen 10mg three times daily for 1 week	No improvement

	24-hour impedance–pH monitoring	esophageal peristalsis<br><br>Partial symptom–reflux correlation for cough and regurgitation (SI 39%, SAP 100%). Hypersensitive esophagus		
**03.2023 Cardiology**			Antihypertensive treatment with Perindopril and Bisoprolol was replaced with Candesartan, Indapamide and Nebivolol	No improvement
**03.2023 Pneumology**			Macrolide antibiotics (Clarithromycin 500mg twice daily for 10 days)<br>Mucolytic therapy (N-acetylcysteine 600mg once daily for 10 days)<br>Antihistamine therapy (Desloratadine 5mg twice daily for 15 days)<br>Oral corticosteroid therapy (Prednisone 30mg once daily for 7 days, then tapering (2 tablets every 7-14 days)<br>Short-acting β2-agonist bronchodilator (Salbutamol, 2 inhalations daily for 2 months)<br>ICS/LABA combination inhaler (fluticasone propionate + salmeterol, 2 inhalations daily for 2 months)	No improvement
**05.2023 Pneumology**			Short-acting muscarinic antagonist (ipratropium bromide, 6 inhalations daily for 1 month)<br>Inhaled corticosteroid (fluticasone propionate, 2 inhalations daily for 2 months)<br>Mucolytic therapy was reintroduced (N-acetylcysteine 600mg once daily for 10 days, Antihistamine therapy	No improvement

			(Desloratadine 5mg twice daily for 15 days)<br>Oral corticosteroid therapy (Prednisone 20 mg once daily for 7 days, then tapering (1 tablet every 30 days)	
**08.2023 Pneumology**	Chest CT	Unremarkable		
**09.2023 Pneumology**	Autofluorescence bronchoscopy with tracheobronchial aspirate for acid-fast bacilli (AFB) and GeneXpert testing, as well as mycological, cytological, and bacteriological examinations	AFB negative; cytology showed no malignant cells; aerobic and fungal cultures were negative.	ICS/LABA combination inhaler (budesonide + formoterol for 2 months)	No improvement
**11.2023 ENT**	ENT fibroscopic examination	Chronic pharyngitis and laryngitis, chronic hypertrophic rhinitis		
**11.2023 Gastroenterology**	Barium swallow	Small hiatal hernia (approximately 3.5 cm) with mild gastroesophageal reflux		
**11.2023 General surgery**		Surgical intervention not indicated for the hiatal gastric hernia		
**01.2024**	Psychiatry consult	No psychiatric causes of cough		
**02.2024 Pneumology**	Chest X-ray<br>Spirometry	No Rx lesions		

		Normal spirometry: a normal Tiffeneau index (84%), normal forced expiratory volume in the 1st second (FEV1 =2.61L - 117% vs predicted value) and normal forced vital capacity (FVC=3.1L - 110% vs predicted value). Referral for neurology and endocrinology		
**03.2024 Endocrinology**	Thyroid panel	Autoimmune (Hashimoto's) thyroiditis with hypothyroidism	Levothyroxine sodium 25mcg once daily	No improvement
**04.2024 Pneumology**			ICS/LABA combination inhaler (budesonide + formoterol for 2 months) Salbutamol as needed	No improvement
**08.2024 Pneumology**	Chest CT	Unremarkable	Intranasal corticosteroid/antihistamine/decongestant combination spray (beclometasone dipropionate + chlorphenamine + phenylephrine, 1 spray daily, for 7 days) Intranasal corticosteroid (mometasone furoate nasal spray, 2 inhalations daily for 5 days)	No improvement
